# Test-retest reliability of short- and long-term heart rate variability in individuals with spinal cord injury

**DOI:** 10.1038/s41393-023-00935-w

**Published:** 2023-10-02

**Authors:** Arphatsorn Ruangsuphaphichat, Lars Brockmann, Patpiya Sirasaporn, Nuttaset Manimmanakorn, Kenneth J. Hunt, Jittima Saengsuwan

**Affiliations:** 1 Department of Rehabilitation Medicine, Faculty of Medicine, Khon Kaen University, Khon Kaen, Thailand; 2 The Laboratory for Rehabilitation Engineering, Institute for Human Centred Engineering, Bern University of Applied Sciences, Biel, Switzerland

**Keywords:** Predictive markers, Rehabilitation

## Abstract

**Study design:**

Cross-sectional.

**Objectives:**

To investigate test-retest reliability of heart rate variability (HRV) metrics in SCI without restriction of activity over long (24-h) and shorter durations (5-min, 10-min, 1-h, 3-h and 6-h).

**Settings:**

University hospital in Khon Kaen, Thailand.

**Methods:**

Forty-five participants (11 with tetraplegia and 34 with paraplegia) underwent two 24-h recordings of RR-intervals to derive time and frequency HRV metrics. Relative reliability was assessed by intraclass correlation coefficient (ICC) and absolute reliability by coefficient of variation (CV) and Bland–Altman limits of agreement (LoA).

**Results:**

For 5- and 10-min durations, eight of eleven HRV metrics had moderate to excellent reliability (ICC 0.40–0.76); the remaining three were poor (ICC < 0.4). HRV values from 1-h and 3-h durations showed moderate to excellent reliability (ICC of 0.46–0.81), except for 1-h reliability of ULF and TP (ICC of 0.06 and 0.30, respectively). Relative reliability was excellent (ICC of 0.77–0.92) for 6-h and 24-h durations in all HRV metrics. Absolute reliability improved as recording duration increased (lower CVs and narrower LoAs). Participants with high AD risk (SCI level at or above T6) showed lower test-retest reliability of HF and LF values than participants with low AD risk.

**Conclusion:**

Relative reliability of HRV was excellent for 6-h and 24-h. The best absolute reliability values were for 24-h duration. Time-domain outcomes were more reliable than frequency domain outcomes. Participants with high risk of AD, particularly those with tetraplegia, showed lower reliability, especially for HF and LF.

## Introduction

Heart rate variability (HRV) is a physiological phenomenon characterising variation in the time interval between consecutive R waves (RR-intervals). It is considered to be intricately modulated by several mechanisms including respiration, thermoregulation, hormonal activity, and the interaction of the sympathetic and parasympathetic divisions of the autonomic nervous system [1]. HRV may be considered a useful predictive marker for diverse adverse clinical outcomes. Low HRV is associated with increased mortality after myocardial infarction [2, 3], increased ICU mortality [4], poor prognosis after traumatic injury [5], or in multiple organ dysfunction in patients with sepsis [6]. It is even considered to be one of the vital signs [5].

HRV can be analysed in the time domain, the frequency domain, and using non-linear methods [1, 7], whereby time and frequency domain analyses are most common in the literature. Time domain analysis is normally reported as standard deviation of all normal-to-normal R-R intervals (SDNN) and root mean square of successive differences between normal heartbeats (RMSSD). Frequency-domain power spectral density analysis can be used to study cardiac autonomic balance [8]. The power spectrum of HRV consists of four components i.e., high frequency (HF) (0.15–0.4 Hz), low frequency (LF) (0.04–0.15 Hz), very low frequency (VLF) (0.0033–0.04 Hz) and ultra-low frequency (ULF) (<0.0033 Hz). HF power has been proposed to be a marker of parasympathetic activity but there is disagreement in respect of the LF component–some studies suggest that LF power reflects sympathetic activity [8, 9], but others propose that LF power reflects both sympathetic and parasympathetic activity as well as baroreflex activity [1, 7]. Nevertheless, the interaction between the sympathetic and parasympathetic divisions is complex and can be modified by multiple stimuli [1]. VLF HRV is thought to be generated intrinsically from the heart and afferent activity of the sympathetic nervous system which is more highly activated with physical activity, while stress may modulate its amplitude and frequency [1, 10]. ULF HRV is thought to be due to very slow-acting biological processes such as circadian rhythms [11].

Spinal cord injury (SCI) leads to an imbalance in cardiogenic autonomic control. This change leads to various complications such as autonomic dysreflexia, arrhythmia and orthostatic hypotension [12, 13]. Moreover, cardiovascular disease is a major problem that leads to morbidity and mortality in individuals with long term SCI [14]. Previous studies have shown that HRV is altered following SCI; for example, LF power was lower in individuals after SCI compared to abled-bodied persons [15–17]. Additionally, persons with paraplegia with a sedentary lifestyle had lower HRV than those with active lifestyles [15]. These findings support the concept that HRV may provide additional objective information about cardiovascular risk and may help to raise awareness of the importance of a healthy lifestyle.

Previous HRV studies showed promising results with good to excellent reliability in able-bodied subjects or in patients after myocardial infarction [18–20]. Considering that reliability is not a fixed characteristic of the variable being measured, but depends on the characteristics of the individuals under investigation [21], it is necessary to determine the reliability of HRV in patients with SCI before HRV can be considered as a practical outcome measure in SCI. Although studies have previously been done to investigate reproducibility of HRV in individuals with SCI, the measurement time was limited to 5–10 min [22, 23]; these durations are simply too short to allow estimation of ULF power. Additionally, taking into consideration that wearable HR monitoring technology is now within reach by everyone, data collected in normal daily conditions without any restrictions on activity may be useful for future analysis.

The aim of this study was to investigate test-retest reliability of HRV metrics in individuals with SCI with no restrictions on activity over a long duration (24 h) and with sub-analysis of shorter durations of measurement (5-min, 10-min, 1-h, 3-h and 6-h).

## Methods

### Subjects

We studied individual with SCI who were admitted at Srinagarind Hospital, which is the largest public hospital in the Northeast region of Thailand, from October 2019 to August 2020. Inclusion criteria were SCI more than 3 months and age ≥18 years. Exclusion criteria were abnormal breathing pattern (respiratory rate >20 breaths/min or <10 breaths/min), fever (body temperature ≥37.8 °C), concomitant cardiac disease as well as endocrine disorders including diabetes mellitus and thyroid disease. Ethical approval for this study was obtained from the Khon Kaen University Committee for Ethics in Human Research (ref. HE621279). Written informed consent was obtained from all participants before the study. The study participants were admitted for annual urological surveillance which is generally composed of urodynamics study or bedside cystometry, ultrasound of the urological system, and voiding cystourethrography. 24-h HRV recordings were done following admission on the day prior to urological check-up. Because every participant was in the same inpatient setting, all participants had a similar daily routine: get up at 5.30–6.00 am, meals served at 8.00 am, 12.00 am and 4.00 pm. The light was turned off for bedtime at 9.00 pm. During the hospitalisation, the patients would have both physical therapy and occupational therapy sessions but during their free time they could do what they want such as going around in the hospital area or staying in bed.

### Study protocol

HRV was measured over a period of 24 h starting at approximately 8 am. Individuals were required to refrain from smoking, and from drinking caffeine or alcohol for 24 h before the study. They were instructed to perform their normal daily, physical activities as usual. Each measurement session was separated by at least 24 h.

### HRV measurements

Raw RR intervals were obtained using a wearable heart rate monitor comprising a wrist watch receiver (Polar V800; Polar Electro Oy, Kempele, Finland) and chest belt sensor (Polar H10). Data recorded from the 24-h measurements were used for a 24-h test-retest reliability analysis. Additionally, HRV outcomes were analysed from recording durations on sub-intervals of 1, 3 and 6 h from specified periods each day (9 am–10 am, 9 am–12 noon and 9 am–3 pm) to determine the shorter duration inter-day test-retest reliability. The 5-min and 10-min segments to be used for short-term analysis were obtained from 9 am–9.05 am and 9 am–9.10 am.

### Outcomes and data processing

Following each measurement, the raw RR intervals stored in the V800 receiver were uploaded to the Polar Flow application, and then exported as a text file to custom-written HRV analysis software implemented in Matlab (The Mathworks, Inc., USA). Some recordings were deemed invalid because of poor signal quality. The remaining data sets were preprocessed for artefact detection and removal. Artefact detection was performed using two methods: (i) maximal and minimal values for plausible RR values were defined (min = 400 ms; max = 1650 ms), (ii) the difference between two successive RR intervals was set to be at a maximum of ±20% of the previous value. For the removal of the detected artefacts, special care was taken not to add new information to the original data sets by removing any artificially introduced combinations of two successive RR intervals from the analysis.

The outcome parameters consisted of both time domain and frequency domain parameters. In the time domain, the HRV metrics SDNN and RMSSD were computed. For the frequency-domain analysis, power in the ULF, VLF, LF, and HF frequency bands was calculated, together with total power (TP). The Lomb-Scargle least squares spectral analysis method for spectral density estimation was used, as it is specifically designed and optimised for non-uniformly spaced data sets such as RR time series. A recent review provides a systematic analysis of the applicability of Lomb-Scargle to in the clinical HRV analysis setting [24].

### Statistical analysis

Continuous parameters are presented as medians (with 25th and 75th percentiles) because the data are not normally distributed. Wilcoxon signed-rank tests were used to test paired differences of the repeated measurements among each participant with significance level set to α = 0.05. Relative test-retest reliability was analysed using the intraclass correlation coefficient ICC_3,1_ and is presented as ICC and 95% confidence interval (CI). ICC ≥ 0.75 represents excellent reliability, ICC < 0.4 is poor reliability and ICC between these ranges is regarded as moderate to good reliability [25]. Absolute reliability was evaluated using the coefficient of variation (CV) [26] and Bland–Altman limits of agreement (LoA) [27]. When the data were heteroscedastic, the data were analysed using log-transformation. The LoA were then back transformed and are presented as ±bx̄, where x̄ is the mean and b is the slope of the LoA [28]. The statistical analyses were perform using SPSS (IBM SPSS Statistics for Windows, Version 28.0. Armonk, NY: IBM Corp).

## Results

Seventy-two individuals participated in this study. During HRV data processing, some data sets were deemed invalid, leading to data from 45 individuals for further analysis. In 17 participants with tetraplegia (34 HRV recordings, 17 data pairs), 6 data pairs were invalid (some single data were valid but because the other was invalid the pair had to be excluded). These were due to 7 inadequate signal durations, 6 noisy signals and 1 signal gap (some data had more than one problem). In 55 participants with paraplegia (110 HRV recordings, 55 data pairs), 21 data pairs were excluded due to 12 noisy signals, 11 inadequate signal durations, 7 multiple skipped heart rate measurements and 3 signal gaps (Supplementary Material 1). Our study had 11 participants with tetraplegia and 34 participants with paraplegia; 71% were male. The mean age was 48.6 years and the median duration after SCI was 5 years (Table 1).

**Table 1:** Demographic data of participants with SCI (*n* = 45).

Variables	*n* (%)
Sex	
Male	32 (71.1)
Female	13 (28.9)
Age (years), Mean (SD)	48.6 (16.9)
Range (years)	18–76
SCI level	
*Tetraplegia (C1-C8):*	
Complete (AIS A)	0 (0)
Incomplete (AIS BCD)	11 (24.4)
*Paraplegia (T1-S5):*	
Complete (AIS A)	11 (24.4)
Incomplete (AIS BCD)	23 (51.1)
SCI level	
*At or above T6 (C1-T6):*	
Complete (AIS A)	2 (4.4)
Incomplete (AIS BCD)	16 (35.6)
*Below T6 (T7-S5):*	
Complete (AIS A)	9 (20.0)
Incomplete (AIS BCD)	18 (40.0)
Cause of SCI	
*Trauma*	19 (42.2)
*Non-Traumatic*	
Degenerative	8 (17.8)
Acquired abnormalities: vascular, inflammatory	10 (22.2)
Neoplastic	5 (11.1)
Infection	3 (6.7)
Duration of SCI (years), median (p25, p75)	5 (3, 9)
Range (years)	0.3–28
Underlying diseases	
No underlying disease	32 (71.1)
Hypertension	7 (15.6)
Dyslipidemia	8 (17.8)
Depression	1 (2.2)
Others	4 (8.9)
Medications	
Anticholinergics	
Oxybutynin	23 (51.1)
Trospium	9 (20.0)
Detrusitol	1 (2.2)
Medications for neuropathic pain	
Gabapentin/Pregabalin	16 (35.6)
Carbamazepine	1 (2.2)
Tricyclic antidepressants	3 (6.7)
Antispastic drug	
Baclofen	14 (31.1)
Clonazepam/Diazepam	10 (22.2)
Tizanidine	3 (6.7)
Antihypertensive drug	
Alpha-blockers	7 (15.6)
Calcium channel blockers	6 (13.3)
Angiotensin-converting enzyme (ACE) inhibitor	2 (4.4)
Beta-blockers	2 (4.4)
Antidepressants	
Fluoxetine	1 (2.2)

*AIS* ASIA Impairment Scale, *P25* 25^th^ percentile, *p75* 75^th^ percentile, *SCI* spinal cord injury, *SD* standard deviation.

There were no statistically significant differences (*p* > 0.05) in any pairs of HRV values for any recording duration (5-min, 10-min, 1-h, 3-h, 6-h and 24-h) except for LF for the 10-min duration (Table 2).

**Table 2:** Test-retest reliability of HRV for each duration (5-min, 10-min, 1-h, 3-h, 6-h and 24-h) in all participants (*n* = 45).

	Day 1, median (p25, p75)	Day 2, median (p25, p75)	p-value	ICC (95%CI)	CV	95% LoA
5-min						
SDNN (ms)	45.0 (29.8, 56.7)	40.7 (30.1, 59.7)	0.71	0.34 (0.05–0.57)	48.8	±1.01 x̄
RMSSD (ms)	17.0 (10.4, 30.6)	16.9 (8.8, 25.4)	0.57	0.72 (0.54–0.83)	43.7	±0.93 x̄
HF (ms^2^)	129.2 (52.9, 395.6)	106.8 (44.5, 405.2)	0.69	0.54 (0.30–0.72)	140.2	±1.68 x̄
LF (ms^2^)	487.4 (205.0, 773.7)	277.9 (135.3, 780.6)	0.45	0.40 (0.13–0.62)	104.6	±1.51 x̄
TP (ms^2^)	638.6 (236.2, 1105.9)	418.3 (196.2, 980.7)	0.96	0.48 (0.22–0.68)	100.4	±1.49 x̄
10-min						
SDNN (ms)	51.6 (33.1, 68.1)	45.7 (33.9, 67.3)	0.40	0.43 (0.15–0.64)	49.2	±1.01 x̄
RMSSD (ms)	16.4 (9.8, 30.4)	17.7 (9.0, 24.9)	0.79	0.76 (0.60–0.86)	40.6	±0.89 x̄
HF (ms^2^)	143.7 (56.8, 334.8)	130.0 (61.1, 364.4)	0.63	0.65 (0.45–0.79)	132.1	±1.65 x̄
LF (ms^2^)	393.7 (208.1, 663.4)	297.2 (172.0, 603.6)	0.037	0.60 (0.37–0.76)	93.1	±1.43 x̄
VLF (ms^2^)	1704.1 (774.9, 3269.5)	1345.6 (618.4, 2529.6)	0.50	0.16 (−0.17–0.43)	144.1	±1.69 x̄
TP (ms^2^)	2500.8 (1091.4, 4693.1)	1818.5 (944.1, 4429.3)	0.39	0.22 (−0.08–0.48)	125.5	±1.62 x̄
1-h						
SDNN (ms)	61.8 (48.3, 89.0)	64.0 (44.1, 88.6)	0.60	0.46 (0.19–0.66)	32.0	±0.74 x̄
RMSSD (ms)	17.3 (10.3, 29.3)	18.9 (9.5, 25.1)	0.81	0.74 (0.58–0.85)	40.6	±0.89 x̄
HF (ms^2^)	146.7 (70.2, 375.3)	166.3 (111.9, 338.8)	0.48	0.60 (0.37–0.76)	99.2	±1.49 x̄
LF (ms^2^)	316.4 (143.4, 712.4)	300.9 (175.3, 622.2)	0.66	0.76 (0.61–0.86)	67.8	±1.24 x̄
VLF (ms^2^)	1120.0 (734.8, 1900.1)	1192.7 (702.6, 2479.6)	0.31	0.64 (0.43–0.79)	56.3	±1.11 x̄
ULF (ms^2^)	2184.2 (1093.5, 4995.2)	2037.2 (1305.2, 4974.2)	0.90	0.06 (−0.23–0.35)	123.2	±1.62 x̄
TP (ms^2^)	4161.4 (2632.4, 7979.5)	4258.2 (2029.6, 8631.4)	0.41	0.30 (0.01–0.54)	75.1	±1.31 x̄
3-h						
SDNN (ms)	78.3 (62.0, 109.7)	74.6 (61.9, 98.5)	0.50	0.72 (0.54–0.84)	23.1	±0.56 x̄
RMSSD (ms)	19.0 (11.3, 28.2)	16.6 (9.6, 26.4)	0.72	0.77 (0.62–0.87)	35.7	±0.80 x̄
HF (ms^2^)	213.5 (105.9, 372.0)	126.9 (77.6, 479.0)	0.70	0.60 (0.37–0.76)	94.9	±1.46 x̄
LF (ms^2^)	298.9 (195.2, 660.2)	267.9 (153.2, 584.7)	0.86	0.81 (0.68–0.89)	64.4	±1.20 x̄
VLF (ms^2^)	1135.9 (661.1, 2036.7)	989.7 (672.3, 1943.9)	0.79	0.77 (0.62–0.87)	52.7	±1.06 x̄
ULF (ms^2^)	5196.8 (2492.1, 9084.4)	4177.5 (2243.1, 7548.5)	0.33	0.70 (0.51–0.82)	76.2	±1.31 x̄
TP (ms^2^)	7111.8 (3904.4, 12474.0)	5955.0 (3964.4, 9925.8)	0.44	0.74 (0.57–0.85)	53.3	±1.07 x̄
6-h						
SDNN (ms)	84.1 (66.3, 107.2)	79.1 (64.1, 109.8)	0.30	0.83 (0.70–0.90)	17.0	±0.43 x̄
RMSSD (ms)	16.8 (10.8, 30.6)	15.6 (10.4, 26.8)	0.50	0.89 (0.81–0.94)	25.7	±0.62 x̄
HF (ms^2^)	166.8 (90.2, 386.0)	183.0 (85.8, 401.4)	0.77	0.77 (0.62–0.87)	52.9	±1.07 x̄
LF (ms^2^)	259.0 (178.7, 697.5)	270.1 (162.5, 669.9)	1.00	0.88 (0.79–0.93)	40.6	±0.89 x̄
VLF (ms^2^)	1151.6 (568.9, 2010.3)	940.1 (611.5, 1694.6)	0.87	0.86 (0.76–0.92)	39.3	±0.87 x̄
ULF (ms^2^)	5903.0 (3393.1, 8765.0)	4787.6 (2901.3, 8571.6)	0.35	0.81 (0.67–0.89)	53.1	±1.06 x̄
TP (ms^2^)	7572.6 (4408.3, 11929.8)	6291.5 (4102.5, 12296.8)	0.37	0.84 (0.73–0.91)	38.9	±0.85 x̄
24-h						
SDNN (ms)	101.7 (81.4, 130.3)	109.0 (82.2, 126.4)	0.76	0.82 (0.69–0.90)	15.7	±0.40 x̄
RMSSD (ms)	20.6 (12.9, 35.2)	22.1 (11.6, 32.5)	0.33	0.91 (0.85–0.95)	14.9	±0.39 x̄
HF (ms^2^)	283.1 (130.3, 656.2)	318.6 (154.2, 519.7)	0.80	0.80 (0.66–0.88)	42.4	±0.91 x̄
LF (ms^2^)	324.1 (211.2, 835.9)	381.9 (239.7, 839.4)	0.97	0.89 (0.80–0.94)	30.4	±0.71 x̄
VLF (ms^2^)	1298.6 (721.6, 2108.5)	1197.6 (695.0, 1766.3)	0.47	0.92 (0.86–0.95)	21.0	±0.52 x̄
ULF (ms^2^)	8549.3 (5405.0, 14469.9)	10046.0 (5235.0, 14171.0)	0.88	0.82 (0.69–0.89)	42.5	±0.92 x̄
TP (ms^2^)	10598.9 (6821.3, 17380.4)	11745.3 (6949.5, 17532.1)	0.94	0.84 (0.73–0.91)	34.8	±0.79 x̄

*CI* confidence interval, *CV* coefficient of variation, *HF* high frequency power, *ICC* intraclass correlation coefficient, *LF* low frequency power, *LoA* limits of agreement, *RMSSD* root mean square of successive differences between normal heartbeats, *SDNN* standard deviation of all normal-to-normal R-R intervals, *TP* total power, *ULF* ultra-low frequency power, *VLF* very low frequency power, *x̄* data mean.

### Relative reliability

**Fig. 1:**
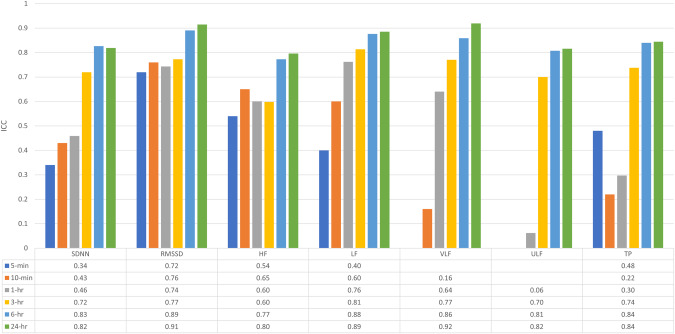
ICCs of each HRV parameter. SDNN, RMSSD, HF, LF, VLF, ULF and TP are shown for each time interval (5-min, 10-min, 1-h, 3-h, 6-h and 24-h) in participants with SCI (*n* = 45).

HRV values for the 5-min duration showed poor reliability of SDNN (ICC of 0.34) and moderate to good reliability of RMSSD, HF, LF and TP (ICC of 0.40–0.72). The 10-min duration showed poor reliability in VLF and TP (ICC of 0.16 and 0.22), moderate to good reliability in SDNN, HF and LF (ICC of 0.43–0.65), and excellent reliability in RMSSD (0.76). HRV outcomes from the 1-h duration showed excellent reliability for LF (ICC = 0.76), moderate to good reliability for SDNN, RMSSD, HF and VLF (ICC of 0.46–0.74), but ULF and TP showed poor reliability (ICC of 0.06 and 0.30, respectively). ULF and TP did however demonstrate markedly increased reliability for 3-h duration (ICC of 0.70 and 0.74, respectively). Relative reliability was excellent (ICC of 0.77–0.92) in all HRV parameters for the 6-h and 24-h durations (Table 3 and Fig. 1).

**Table 3:** Summary of test-retest reliability of heart rate variability for each duration (5-min, 10-min, 1-h, 3-h, 6-h and 24-h) in all participants (*n* = 45).

	SDNN (ms)	RMSSD (ms)	HF (ms^2^)	LF (ms^2^)	VLF (ms^2^)	ULF (ms^2^)	TP (ms^2^)
ICC (95% CI)							
5-min	0.34 (0.05–0.57)	0.72 (0.54–0.83)	0.54 (0.30–0.72)	0.40 (0.13–0.62)	NA	NA	0.48 (0.22–0.68)
10-min	0.43 (0.15–0.64)	0.76 (0.60–0.86)	0.65 (0.45–0.79)	0.60 (0.37–0.76)	0.16 (−0.17–0.43)	NA	0.22 (−0.08–0.48)
1-h	0.46 (0.19–0.66)	0.74 (0.58–0.85)	0.60 (0.37–0.76)	0.76 (0.61–0.86)	0.64 (0.43–0.79)	0.06 (−0.23–0.35)	0.30 (0.01–0.54)
3-h	0.72 (0.54–0.84)	0.77 (0.62–0.87)	0.60 (0.37–0.76)	0.81 (0.68–0.89)	0.77 (0.62–0.87)	0.70 (0.51–0.82)	0.74 (0.57–0.85)
6-h	0.83 (0.70–0.90)	0.89 (0.81–0.94)	0.77 (0.62–0.87)	0.88 (0.79–0.93)	0.86 (0.76–0.92)	0.81 (0.67–0.89)	0.84 (0.73–0.91)
24-h	0.82 (0.69–0.90)	0.91 (0.85–0.95)	0.80 (0.66–0.88)	0.89 (0.80–0.94)	0.92 (0.86–0.95)	0.82 (0.69–0.89)	0.84 (0.73–0.91)
CV (%)							
5-min	48.8	43.7	140.2	104.6	NA	NA	100.4
10-min	49.2	40.6	132.1	93.1	144.1	NA	125.5
1-h	32.0	40.6	99.2	67.8	56.3	123.2	75.1
3-h	23.1	35.7	94.9	64.4	52.7	76.2	53.3
6-h	17.0	25.7	52.9	40.6	39.3	53.1	38.9
24-h	15.7	14.9	42.4	30.4	21.0	42.5	34.8
95% LoA							
5-min	±1.01 x̄	±0.93 x̄	±1.68 x̄	±1.51 x̄	NA	NA	±1.49 x̄
10-min	±1.01 x̄	±0.89 x̄	±1.65 x̄	±1.43 x̄	±1.69 x̄	NA	±1.62 x̄
1-h	±0.74 x̄	±0.89 x̄	±1.49 x̄	±1.24 x̄	±1.11 x̄	±1.62 x̄	±1.31 x̄
3-h	±0.56 x̄	±0.80 x̄	±1.46 x̄	±1.20 x̄	±1.06 x̄	±1.31 x̄	±1.07 x̄
6-h	±0.43 x̄	±0.62 x̄	±1.07 x̄	±0.89 x̄	±0.87 x̄	±1.06 x̄	±0.85 x̄
24-h	±0.40 x̄	±0.39 x̄	±0.91 x̄	±0.71 x̄	±0.52 x̄	±0.92 x̄	±0.79 x̄

*CI* confidence interval, *CV* coefficient of variation, *HF* high frequency power, *ICC* intraclass correlation coefficient, *LF* low frequency power, *LoA* limits of agreement, *NA* Not applicable, *RMSSD* root mean square of successive differences between normal heartbeats, *SDNN* standard deviation of all normal-to-normal R-R intervals, *TP* total power, *ULF* ultra-low frequency power, *VLF* very low frequency power, *x̄* data mean.

### Absolute reliability

**Fig. 2:**
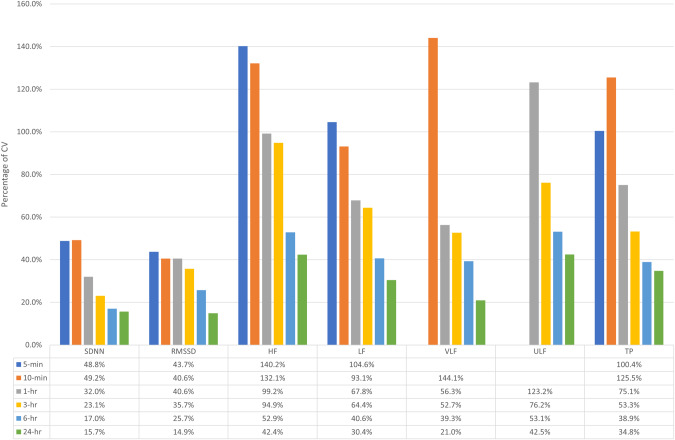
Coefficient of variation of each HRV parameter. SDNN, RMSSD, HF, LF, VLF, ULF and TP are shown for each time interval (5-min, 10-min, 1-h, 3-h, 6-h and 24-h) in participants with SCI (*n* = 45).

**Fig. 3:**
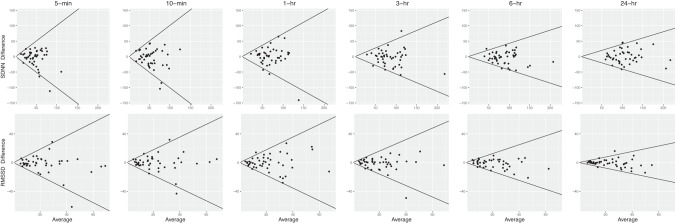
Bland–Altman plot. Mean differences and 95% limits of agreement (LoA) among time domain HRV measures (SDNN and RMSSD) in 5-min, 10-min, 1-h, 3-h, 6-h and 24-h in participants with SCI (*n* = 45). The diagonal lines represent the 95% LoA.

**Fig. 4:**
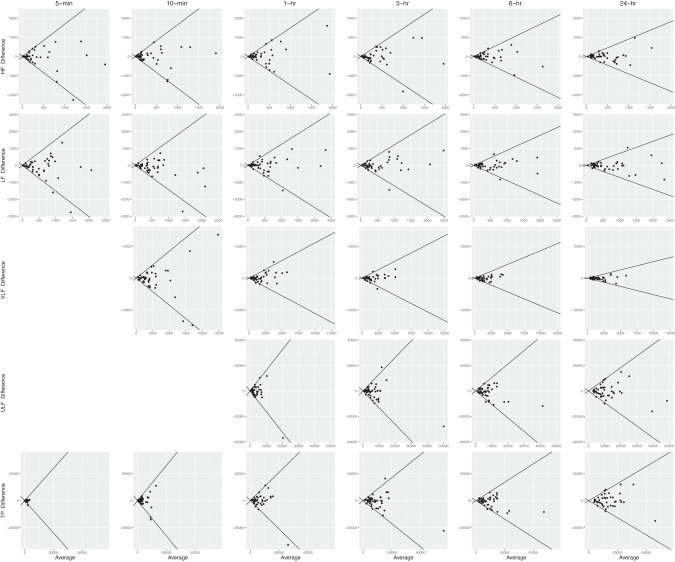
Bland–Altman plot. Mean differences and 95% LoA among frequency domain HRV measures (HF, LF, VLF, ULF and TP) in 5-min, 10-min, 1-h, 3-h, 6-h and 24-h in participants with SCI (*n* = 45). The diagonal lines represent the 95% LoA.

Better absolute reliability was found for longer durations. CVs were in the range of 40.6–144.1% for HRV values of 5-min and 10-min duration and decreased to 14.9–42.5% for the 24-h duration. Generally, CV decreased by more than half in all recorded HRV parameters towards the 24-h duration (Fig. 2). There was better CV in the time domain outcomes compared to frequency domain outcomes (Table 3). Overall, Bland–Altman plots showed narrower limits of agreement in all HRV parameters as the observation period increased (Figs. 3 and 4).

### Test-retest reliability across groups based on risk of autonomic dysreflexia

Eighteen participants were in the high AD risk group (SCI level at or above T6). There were no significant differences between any pairs of HRV values for any duration in either group. Participants with high AD risk showed lower test-retest reliability of all HRV metrics compared to participants with low AD risk for the 5-min and 10-min durations. Additionally, they had lower test-retest reliability of HF and LF values compared to participants with low AD risk for all durations. The ICCs of HF were 0.31, 0.43, 0.43, 0.26, 0.59 and 0.66 for participants with SCI level at or above T6, while the ICCs were 0.84, 0.84, 0.76, 0.82, 0.87 and 0.89 for participants with lesion below T6 (for the 5-min, 10-min, 1-h, 3-h, 6-h and 24-h durations, respectively). ICCs of LF were 0.23, 0.32, 0.36, 0.58, 0.75 and 0.82 for participants with SCI level at or above T6 while ICCs were 0.64, 0.81, 0.90, 0.92, 0.94 and 0.95 for participants with lesion below T6. For these outcomes, it is clear that participants with lesion level at or above T6 had lower reliability for all durations. However, participants with a lesion level at or above T6 showed better relative reliability in SDNN, ULF and TP for the 3-h, 6-h and 24-h durations. HF and ULF were the least reliable HRV outcomes based on the lowest absolute reliability in both groups. The highest absolute reliability, as classified by smallest CV and narrowest limits of agreement were in the time domain metrics and for the 24-h duration in both groups. The CVs were 12.9% and 13.6% for SDNN and RMSSD in participants with SCI level at or above T6, and 17.3% and 15.7% in participants with SCI level below T6 (Supplementary Material 2, Tables 1 and 2).

### Test-retest reliability across groups based on tetraplegia or paraplegia

The group of persons with paraplegia (*n* = 34) showed a significant difference in LF in the 10-min pair (*p* = 0.022). Persons with tetraplegia (*n* = 11) showed a significant difference between ULF pairs for the 1-h duration (*p* = 0.007). Participants with tetraplegia demonstrated lower relative test-retest reliability than those with paraplegia in most HRV metrics for every duration. For example, participants with tetraplegia exhibited lower test-retest reliability of HF and LF than participants with paraplegia: the ICCs of HF were 0.34, 0.50, 0.53, 0.20, 0.57 and 0.51 for participants with tetraplegia, while the ICCs were 0.67, 0.72, 0.66, 0.81, 0.86 and 0.89 for participants with paraplegia (for the 5-min, 10-min, 1-h, 3-h. 6-h and 24-h durations, respectively). The ICCs of LF were 0.17, 0.41, 0.73, 0.37, 0.64 and 0.66 for participants with tetraplegia, while the ICCs were 0.45, 0.60, 0.77, 0.92, 0.93 and 0.93 for participants with paraplegia. Thus, the group with tetraplegia had lower reliability of these outcomes for all durations. The highest absolute reliability as classified by lowest CV and narrowest limits of agreement were in the time domain metrics and for the 24-h duration in both groups (CVs of 13.3 % and 11.6 % for SDNN and RMSSD in participants with tetraplegia, and CVs of 16.4 % and 15.9 % in participants with paraplegia (Supplementary Material 2, Tables 3 and 4).

## Discussion

This study aimed to investigate test-retest reliability of HRV metrics in individuals with SCI with no restrictions on activity over a long duration (24-h) and with sub-analysis of shorter durations of measurement (5-min, 10-min, 1-h, 3-h and 6-h). Based on ICC value ranging from 0.77 to 0.92, excellent relative reliability was found in all HRV parameters derived from 6-h and 24-h periods. Overall, the time-domain parameters were more reliable than the frequency domain parameters.

Regarding 5-min HRV metrics, La Fountaine et al., conducted a test-retest reliability study of HRV in seven participants with tetraplegia in the supine position and found moderate to good relative reliability of HF and LF (ICC of 0.66 and 0.44) [23]. Our study also showed that HF exhibited better relative test-retest reliability compared to LF for the 5-min duration, but our ICC values (ICC of 0.34 and 0.17 for HF and LF) were lower than those reported in the previous study. The difference in position and activity of participants may have played a role in this discrepancy. Ditor et al. [22] examined the test-retest reliability of 10-min HRV and found that the reliability of HF was poorer than LF, the ICCs for HF and LF were 0.53 and 0.84 in ten participants with SCI (all levels), and the ICC of HF and LF were 0.66 and 0.82 in six participants with tetraplegia. In contrast to the results of Ditor et al., we found better relative reliability of HF compared to LF for the 10-min duration. The ICCs of HF and LF in the overall SCI population were 0.65 and 0.60, and the ICCs of HF and LF were 0.50 and 0.41 in participants with tetraplegia. There seems to be conflicting results among studies regarding whether LF or HF showed better relative reliability, so this may need to be interpreted with caution [22, 23]. In our study, however, it should be noted that LF showed better absolute reliability, as evidenced by a smaller CV and narrower LoA compared to HF.

The reliability of long-term duration was comparable to previous studies in healthy individuals, patients with coronary disease and patients with hypertensive disease, which showed moderate to very good correlations of 0.60 and 0.98 [29, 30]. Those authors gave a cautionary note that some individuals without heart disease had considerably higher day-to-day variation in heart rate variability, so that care should be exercised when interpreting HRV outcomes from healthy individuals [29, 30]. These previous studies explored long term recording of HRV, but they used only correlation analysis and other reliability outcomes such as ICC or CV were not reported.

Regarding the relative reliability, different ranges of ICC are well defined and recognised for interpretation as poor, moderate to good, or excellent. However, CV and LoA were interpreted differently among previous studies. For example, the CV regarded as good reliability varied among studies in the range 10–30% [31, 32]. Based on the ICC, together with the CV and LoA, we found that the reliability for long term measurement was excellent especially for time domain parameters compared to frequency domain parameters. This finding was consistent with previous studies in patients with cardiac disease, patients with hypertension as well as in able-bodied subjects [18, 29, 33, 34].

The CV for 24-h recording of HRV observed in individuals with SCI in this study (14.9–42.5%) was comparable to those of healthy subjects (6–88%), but the CV for short-term HRV values in our study of 32–123% was much higher than elsewhere [34, 35]. The finding of lower reliability may have several causes. Firstly, it has been found that in clinical populations HRV is less reliable compared to able-bodied subjects. For example, Lord et al. found CV of LF power of 45% in controls and 76% in heart transplant patients [36]. Secondly, as our subjects have unique cardiogenic autonomic balance, especially those with injury level at or above T6 [37], higher day to day variation can occur. This is demonstrated by the lower test-retest reliability we found in the group with high AD risk and especially in participants with tetraplegia. Additionally, HRV is known to be affected by factors including physical activity level, rate and depth of respiration, postural change and acute psychological factors, as described in previous studies [18, 38–40]; since our study did not limit activity, it is possible that HRV varied more than in controlled conditions.

In general, a data recording period of 24 h is recommended when ULF power is to be analysed [1]. However, in our study, the shortest period that we examine all HRV parameters including ULF power was 1-h. According to the Nyquist-Shannon theorem, in order to gain an adequate waveform to analyse the data, the sampling frequency has to be at least twice the frequency of interest [41]. In practice, however, it is necessary due to measurement noise to increase the sampling rate to at least 10 times the theoretical lower bound. Since the upper frequency bound of ULF is 0.0033 Hz, corresponding to a period of 5 min, a tenfold recording interval of at least approximately one hour is required.

A limitation of our study was the reduced sample size caused by rejection of multiple data sets, caused principally by shifting of the HR chest belt sensor during normal daily activities. The high percentage of invalid recordings implies that 24-h recordings with wearable devices, specifically for the purpose of HRV analysis within the SCI population, is challenging. Care should therefore be exercised by patients and their carers to ensure, as far as possible, that the chest belt remains in position. The data in participants with tetraplegia were mainly excluded due to inadequate signal duration. This may be due to the removal of the HR belt before the proposed time by the patients or their relatives. There were no HRV data from participants with complete tetraplegia. Therefore, the generalisation of the data may be limited in this regard. It should be noted that although medications affect HRV, it should not affect the repeatability of HRV as the patients were taking the same medications every day [42–45].

Future work should focus on improving methods for HR measurement to achieve acceptable reliability in all HRV parameters in a shorter period, e.g. during measurement at rest, with controlled breathing or with limited postural change. The test-retest reliability data were mainly focused on no restriction of activity, thus improvement in reliability in under more-controlled conditions can be expected.

## Conclusions

Relative reliability of HRV was excellent for 6 and 24-h recording durations and the best absolute reliability values were for 24-h recordings. Taking into consideration both relative and absolute reliability, longer-duration recording led to progressively better reliability. Time-domain HRV outcomes were more reliable than frequency domain outcomes. Participants with high risk of AD, particularly those with tetraplegia, showed lower reliability, especially for HF and LF. Additionally, there were challenges in acquiring long-duration recordings using the wearable devices without any restriction in activity in participants with SCI. Care should be taken to ensure that the chest belt remains in position.

### Supplementary information

Reproducibility checklist

Supplementary material 1

Supplementary material 2

## Data Availability

All relevant data are within this manuscript and raw data are archived by the corresponding author.
