# Supplementary material for: Test-retest reliability of short- and long-term heart rate variability in individuals with spinal cord injury
Source: Spinal Cord. 2023 Oct 2;61(12):658–66. doi: 10.1038/s41393-023-00935-w (PMC10691965; doi:10.1038/s41393-023-00935-w)
Supplement: Supplementary file 2 — Supplementary material 1 [file 41393_2023_935_MOESM2_ESM.docx]

Examples of discarded data

- 1. Inadequate signal duration


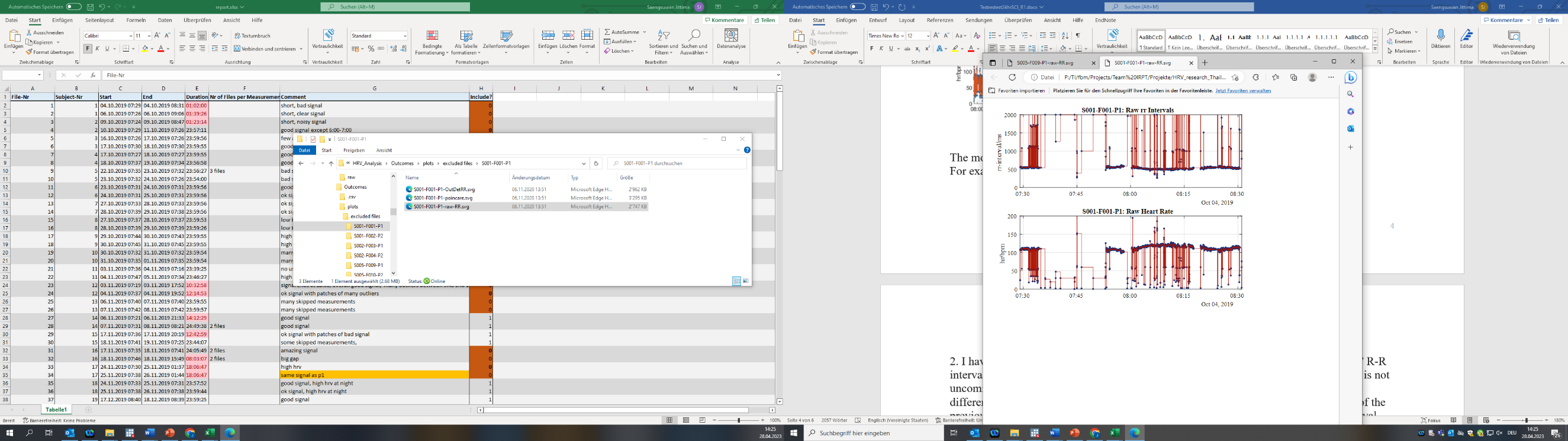


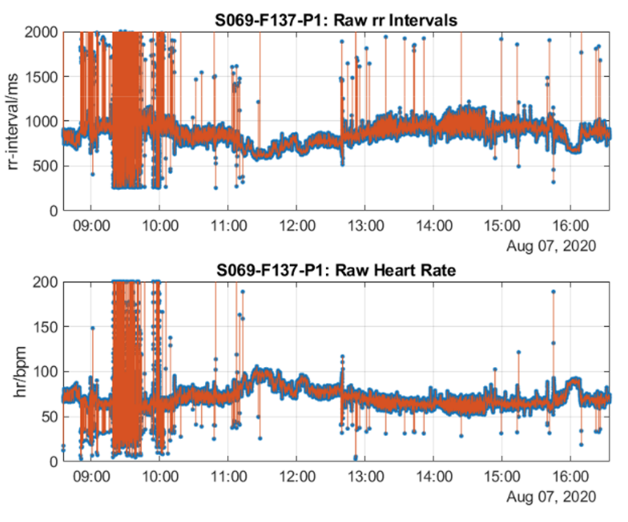


- 1. Noisy signal


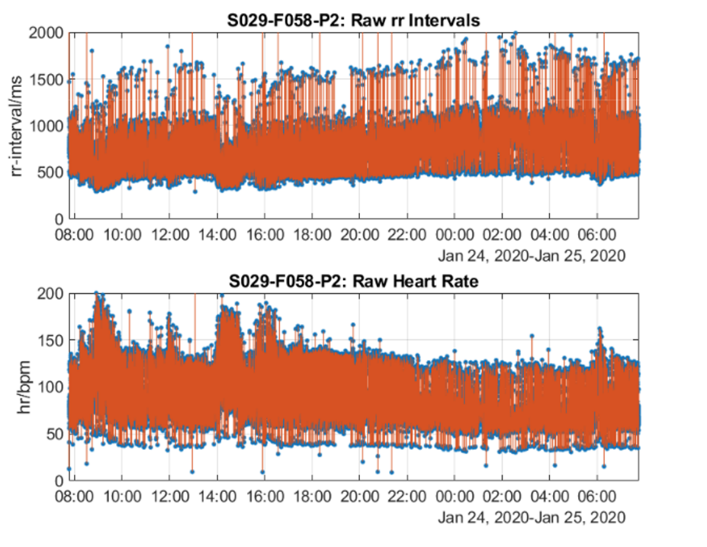


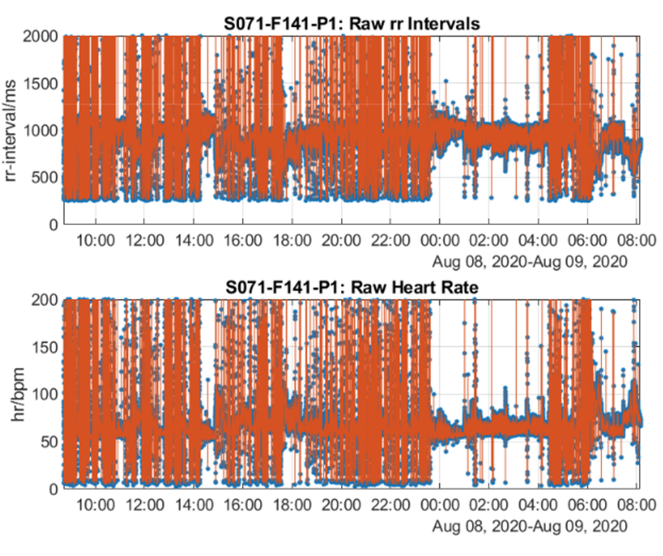


1.3. Signal gap


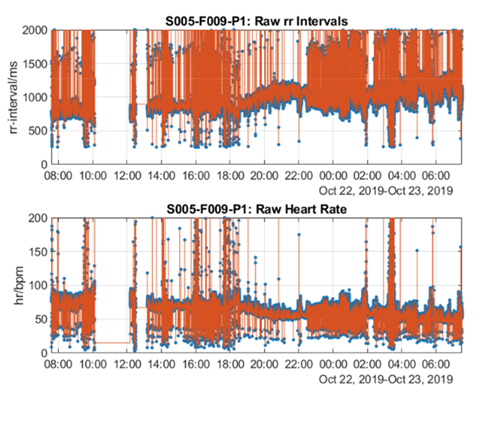


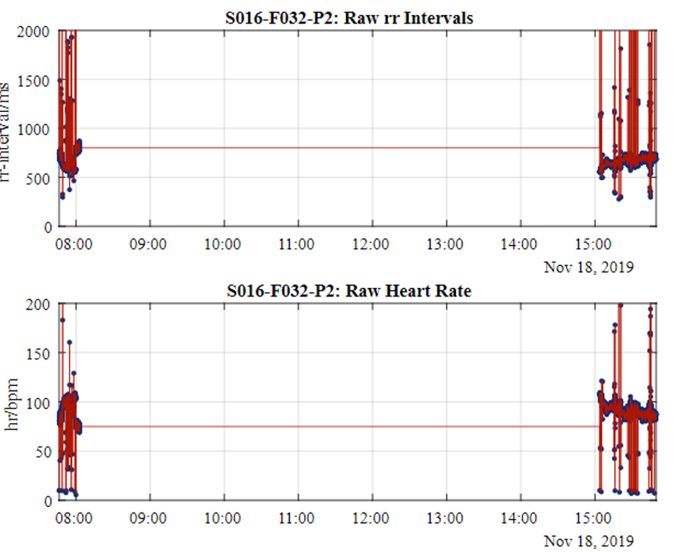


1.4 Multiple skipped heart rate measurements


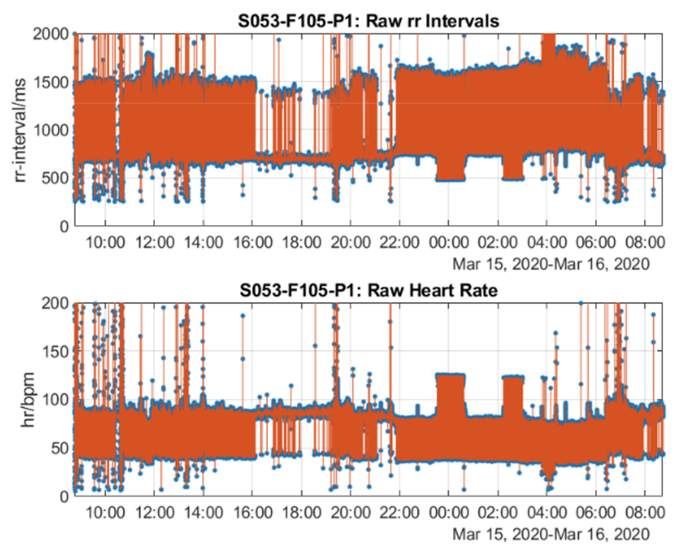


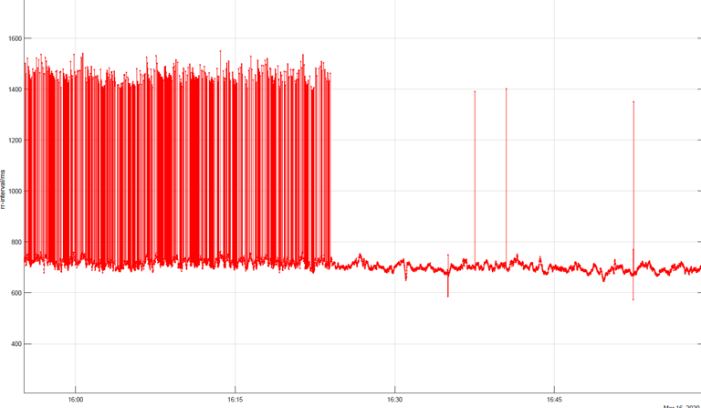
 Zoomed view:
