# Supplementary material for: Test-retest reliability of short- and long-term heart rate variability in individuals with spinal cord injury
Source: Spinal Cord. 2023 Oct 2;61(12):658–66. doi: 10.1038/s41393-023-00935-w (PMC10691965; doi:10.1038/s41393-023-00935-w)
Supplement: Supplementary file 3 — Supplementary material 2 [file 41393_2023_935_MOESM3_ESM.docx]

**Supplementary table 1** Test-retest reliability of HRV for each duration (SCI level at or above T6, n = 18)

|  | Day 1,  median (p25, p75) | Day 2,  median (p25, p75) | p-value | ICC (95%CI) | CV | 95% LoA |
| --- | --- | --- | --- | --- | --- | --- |
| 5-min |  |  |  |  |  |  |
| SDNN (ms) | 50.4 (42.5, 72.0) | 36.9 (27.1, 59.7) | 0.35 | -0.03 (-0.44 - 0.42) | 65.5 | ± 1.16 x̄ |
| RMSSD (ms) | 21.8 (12.9, 35.3) | 18.2 (11.5, 24.2) | 0.13 | 0.59 (0.21 - 0.82) | 47.8 | ± 0.93 x̄ |
| HF (ms^2^) | 262.1 (68.7, 482.7) | 130.0 (68.2, 444.7) | 0.73 | 0.31 (-0.18 - 0.67) | 173.4 | ± 1.77 x̄ |
| LF (ms^2^) | 590.3 (246.3, 861.9) | 310.5 (135.3, 823.0) | 0.25 | 0.23 (-0.22 - 0.61) | 144.4 | ± 1.64 x̄ |
| TP (ms^2^) | 825.4 (532.5, 1562.1) | 432.6 (219.2, 1386.2) | 0.61 | 0.27 (-0.20 - 0.64) | 136.9 | ± 1.63 x̄ |
| 10-min |  |  |  |  |  |  |
| SDNN (ms) | 54.1 (41.2, 72.2) | 42.9 (32.7, 51.9) | 0.17 | 0.16 (-0.24 - 0.55) | 56.6 | ± 0.99 x̄ |
| RMSSD (ms) | 19.0 (13.8, 36.1) | 17.7 (11.8, 24.6) | 0.47 | 0.70 (0.38 - 0.88) | 38.1 | ± 0.82 x̄ |
| HF (ms^2^) | 280.3 (83.4, 463.3) | 144.0 (61.1, 453.2) | 0.80 | 0.43 (0.68 - 0.92) | 118.5 | ± 1.59 x̄ |
| LF (ms^2^) | 511.3 (288.2, 752.0) | 415.7 (172.0, 603.6) | 0.21 | 0.32 (-0.14 - 0.68) | 120.9 | ± 1.56 x̄ |
| VLF (ms^2^) | 1900.7 (994.7, 3269.5) | 1147.4 (545.0, 1937.0) | 0.17 | -0.05 (-0.45 - 0.39) | 176.3 | ± 1.70 x̄ |
| TP (ms^2^) | 2628.9 (1797.2, 4852.1) | 1719.1 (944.2, 2903.5) | 0.25 | -0.01 (-0.42 - 0.43) | 152.0 | ± 1.63 x̄ |
| 1-hr |  |  |  |  |  |  |
| SDNN (ms) | 69.2 (57.2, 92.4) | 64.5 (46.4, 77.8) | 0.47 | 0.30 (-0.15 – 0.66) | 31.9 | ± 0.72 x̄ |
| RMSSD (ms) | 19.2 (14.5, 43.3) | 20.6 (14.4, 25.1) | 0.83 | 0.68 (0.32 – 0.87) | 34.5 | ± 0.80 x̄ |
| HF (ms^2^) | 189.7 (110.2, 548.9) | 199.4 (138.2, 496.5) | 0.23 | 0.43 (-0.04 – 0.74) | 95.6 | ± 1.48 x̄ |
| LF (ms^2^) | 451.8 (225.8, 785.8) | 348.4 (225.8, 622.2) | 0.73 | 0.36 (-0.13 – 0.71) | 76.4 | ± 1.34 x̄ |
| VLF (ms^2^) | 1325.0 (920.0, 2349.5) | 1297.6 (718.2, 1992.1) | 0.73 | 0.70 (0.35 – 0.88) | 38.9 | ± 0.86 x̄ |
| ULF (ms^2^) | 2504.0 (1821.9, 5454.9) | 1648.2 (1070.0, 3067.1) | 0.11 | 0.03 (-0.41 – 0.64) | 121.6 | ± 1.57 x̄ |
| TP (ms^2^) | 4893.0 (3355.7, 9698.7) | 4357.5 (2345.0, 6548.8) | 0.50 | 0.16 (-0.31 – 0.57) | 72.1 | ± 1.25 x̄ |
| 3-hr |  |  |  |  |  |  |
| SDNN (ms) | 85.6 (61.7, 112.7) | 80.7 (63.8, 104.8) | 0.44 | 0.80 (0.56 - 0.92) | 22.6 | ± 0.54 x̄ |
| RMSSD (ms) | 23.7 (16.3, 32.8) | 24.0 (14.9, 27.1) | 0.83 | 0.60 (0.20 – 0.83) | 34.8 | ± 0.79 x̄ |
| HF (ms^2^) | 226.5 (147.7, 603.0) | 309.0 (104.7, 617.0) | 0.35 | 0.26 (-0.25 – 0.65) | 102.4 | ± 1.53 x̄ |
| LF (ms^2^) | 407.5 (195.2, 842.6) | 417.8 (179.8, 1247.8) | 0.64 | 0.58 (0.16 – 0.82) | 73.2 | ± 1.31 x̄ |
| VLF (ms^2^) | 1189.2 (701.2, 2487.4) | 1041.5 (709.0, 2401.8) | 0.58 | 0.75 (0.45 – 0.90) | 39.7 | ± 0.87 x̄ |
| ULF (ms^2^) | 4807.2 (2670.2, 9787.8) | 4407.5 (2195.5, 8392.6) | 0.30 | 0.79 (0.53 – 0.92) | 73.8 | ± 1.26 x̄ |
| TP (ms^2^) | 7822.1 (3829.3, 12840.1) | 6919.1 4225.4, 11827.1) | 0.55 | 0.81 (0.56 – 0.92) | 51.4 | ± 1.03 x̄ |
| 6-hr |  |  |  |  |  |  |
| SDNN (ms) | 85.7 (61.7, 112.7) | 80.7 (63.8, 104.8) | 0.44 | 0.88 (0.71 – 0.95) | 15.6 | ± 0.39 x̄ |
| RMSSD (ms) | 23.7 (16.3, 32.8) | 24.0 (14.9, 27.1) | 0.83 | 0.84 (0.62 – 0.94) | 19.7 | ± 0.48 x̄ |
| HF (ms^2^) | 226.5 (147.7, 603.0) | 309.0 (104.7, 617.0) | 0.35 | 0.59 (0.17 – 0.83) | 53.5 | ± 1.07 x̄ |
| LF (ms^2^) | 407.5 (195.2, 842.6) | 417.8 (179.8, 1247.8) | 0.64 | 0.75 (0.45 – 0.90) | 40.4 | ± 0.89 x̄ |
| VLF (ms^2^) | 1189.2 (701.2, 2487.4) | 1041.5 (709.0, 2401.8) | 0.58 | 0.88 (0.70 – 0.95) | 22.0 | ± 0.53 x̄ |
| ULF (ms^2^) | 4807.2 (2670.2, 9787.8) | 4407.5 (2195.5, 8392.6) | 0.30 | 0.87 (0.69 – 0.95) | 48.4 | ± 1.00 x̄ |
| TP (ms^2^) | 7822.1 (3829.3, 12840.1) | 6919.1 (4225.4, 11827.1) | 0.55 | 0.88 (0.71 – 0.95) | 34.7 | ± 0.78 x̄ |
| 24-hr |  |  |  |  |  |  |
| SDNN (ms) | 111.2 (79.5, 133.7) | 109.2 (87.6, 129.1) | 0.97 | 0.89 (0.74 – 0.96) | 12.9 | ± 0.34 x̄ |
| RMSSD (ms) | 33.0 (17.4, 36.0) | 26.9 (15.3 35.5) | 0.0502 | 0.92 (0.78 – 0.97) | 13.6 | ± 0.33 x̄ |
| HF (ms^2^) | 440.1 (158.8, 757.3) | 493.4 (171.5, 674.8) | 0.50 | 0.66 (0.28 – 0.86) | 37.6 | ± 0.84 x̄ |
| LF (ms^2^) | 680.7 (261.4, 1084.4) | 416.7 (239.7, 1114.1) | 0.28 | 0.82 (0.58 – 0.93) | 36.1 | ± 0.80 x̄ |
| VLF (ms^2^) | 1599.9 (721.6, 2910.8) | 1502.0 (695.0, 2148.8) | 0.11 | 0.91 (0.78 – 0.97) | 20.7 | ± 0.46 x̄ |
| ULF (ms^2^) | 10473.9 (4855.6, 15054.8) | 9731.9 (7075.8, 15922.0) | 0.87 | 0.88 (0.70 – 0.95) | 36.3 | ± 0.83 x̄ |
| TP (ms^2^) | 13418.9 (6326.2, 18311.4) | 12129.2 (7921.4, 17576.9) | 0.77 | 0.89 (0.72 – 0.96) | 29.0 | ± 0.70 x̄ |

SDNN standard deviation of all normal-to-normal R-R intervals; RMSSD root mean square of successive differences between normal heartbeats; HF high frequency; LF low frequency; VLF very low frequency; ULF ultra-low frequency; TP total power; SD standard deviation; CI confidence interval; ICC intraclass correlation coefficient; CV coefficient of variation; LOA limits of agreement; x̄ data mean

**Supplementary table 2** Test-retest reliability of HRV for each duration (SCI level below T6, n = 27)

|  | Day 1,  median (p25, p75) | Day 2,  median (p25, p75) | p-value | ICC (95%CI) | CV | 95% LoA |
| --- | --- | --- | --- | --- | --- | --- |
| 5-min |  |  |  |  |  |  |
| SDNN (ms) | 39.6 (27.7, 51.1) | 41.1 (30.1, 61.0) | 0.15 | 0.68 (0.42 - 0.84) | 35.8 | ± 0.77 x̄ |
| RMSSD (ms) | 14.7 (9.3, 26.4) | 16.8 (8.7, 26.5) | 0.47 | 0.85 (0.70 - 0.93) | 40.9 | ± 0.89 x̄ |
| HF (ms^2^) | 98.8 (47.0, 255.9) | 98.8 (32.9, 317.6) | 0.84 | 0.84 (0.68 - 0.93) | 117.8 | ± 1.59 x̄ |
| LF (ms^2^) | 256.1 (198.6, 656.8) | 277.9 (122.6, 700.9) | 0.99 | 0.64 (0.35 - 0.82) | 76.4 | ± 1.33 x̄ |
| TP (ms^2^) | 369.4 (231.1, 879.8) | 418.3 (169.2, 952.4) | 0.70 | 0.79 (0.59 - 0.90) | 74.5 | ± 1.31 x̄ |
| 10-min |  |  |  |  |  |  |
| SDNN (ms) | 49.3 (30.1, 68.1) | 51.3 (33.9, 71.3) | 0.88 | 0.64 (0.35 - 0.82) | 44.0 | ± 0.93 x̄ |
| RMSSD (ms) | 16.4 (8.8, 24.5) | 19.2 (8.7, 25.3) | 0.26 | 0.81 (0.63 - 0.91) | 42.2 | ± 0.90 x̄ |
| HF (ms^2^) | 119.8 (40.8, 261.2) | 130.0 (61.1, 328.1) | 0.77 | 0.84 (0.68 – 0.92) | 141.0 | ± 1.68 x̄ |
| LF (ms^2^) | 356.3 (186.6, 578.0) | 267.2 (165.7, 653.7) | 0.11 | 0.81 (0.62 - 0.91) | 73.5 | ± 1.30 x̄ |
| VLF (ms^2^) | 1551.3 (681.1, 3498.1) | 1506.7 (618.4, 3223.2) | 0.75 | 0.36 (-0.02 - 0.65) | 122.3 | ± 1.60 x̄ |
| TP (ms^2^) | 2004.4 (877.7, 4690.4) | 1950.6 (905.3, 4716.1) | 0.92 | 0.45 (0.09 - 0.71) | 107.4 | ± 1.53 x̄ |
| 1-hr |  |  |  |  |  |  |
| SDNN (ms) | 60.0 (46.9, 89.0) | 64.0 (44.0, 92.1) | 0.20 | 0.61 (0.32– 0.80) | 32.1 | ± 0.73 x̄ |
| RMSSD (ms) | 16.6 (8.3, 24.7) | 16.8 (7.5, 29.0) | 0.66 | 0.78 (0.57 – 0.89) | 44.4 | ± 0.92 x̄ |
| HF (ms^2^) | 134.3 (41.2, 307.6) | 125.0 (35.2, 316.3) | 0.90 | 0.76 (0.54 – 0.88) | 101.6 | ± 1.51 x̄ |
| LF (ms^2^) | 264.9 (135.0, 626.7) | 251.5 (115.4, 626.3) | 0.95 | 0.90 (0.80 – 0.95) | 61.9 | ± 1.18 x̄ |
| VLF (ms^2^) | 1120.0 (569.5, 1887.1) | 1016.0 (453.6, 3490.4) | 0.13 | 0.62 (0.33 – 0.81) | 66.6 | ± 1.22 x̄ |
| ULF (ms^2^) | 2020.0 (985.1, 4511.8) | 2719.0 (1305.2, 4976.2) | 0.25 | 0.25 (-0.12 – 0.57) | 124.2 | ± 1.60 x̄ |
| TP (ms^2^) | 3917.2 (2236.2, 7980.0) | 4258.3 (1947.8, 9977.4) | 0.11 | 0.58 (0.27 – 0.78) | 77.0 | ± 1.31 x̄ |
| 3-hr |  |  |  |  |  |  |
| SDNN (ms) | 78.3 (63.8, 104.7) | 72.7 (60.1, 94.5) | 0.80 | 0.60 (0.29 - 0.80) | 23.4 | ± 0.58 x̄ |
| RMSSD (ms) | 14.8 (9.9, 25.6) | 15.2 (8.2, 22.3) | 0.79 | 0.88 (0.76 – 0.94) | 36.3 | ± 0.82 x̄ |
| HF (ms^2^) | 171.6 (70.4, 356.1) | 107.7 (47.7, 330.0) | 0.12 | 0.82 (0.64 – 0.91) | 89.8 | ± 1.42 x̄ |
| LF (ms^2^) | 283.3 (195.2, 615.4) | 250.9 (148.8, 485.5) | 0.43 | 0.92 (0.82 – 0.96) | 58.3 | ± 1.13 x̄ |
| VLF (ms^2^) | 955.2 (636.7, 1794.3) | 984.8 (553.3, 1929.6) | 0.66 | 0.79 (0.59 – 0.90) | 60.5 | ± 1.17 x̄ |
| ULF (ms^2^) | 5196.8 (1938.6, 8051.1) | 3919.9 (2396.9, 6681.3) | 0.70 | 0.28 (-0.11 – 0.60) | 77.7 | ± 1.34 x̄ |
| TP (ms^2^) | 7111.9 (4074.7, 12339.9) | 5505.9 (3610.7, 8998.5) | 0.73 | 0.52 (0.18 – 0.75) | 54.5 | ± 1.09 x̄ |
| 6-hr |  |  |  |  |  |  |
| SDNN (ms) | 78.3 (63.8, 104.7) | 72.7 (60.1, 94.5) | 0.80 | 0.71 (0.46 – 0.86) | 17.9 | ± 0.45 x̄ |
| RMSSD (ms) | 14.8 (9.9, 25.6) | 15.2 (8.2, 22.3) | 0.79 | 0.92 (0.90 – 0.98) | 29.2 | ± 0.69 x̄ |
| HF (ms^2^) | 171.6 (70.4, 356.2) | 107.7 (47.7, 330.0) | 0.12 | 0.87 (0.74 – 0.94) | 52.5 | ± 1.06 x̄ |
| LF (ms^2^) | 283.3 (195.2, 615.5) | 250.9 (148.8, 485.5) | 0.43 | 0.94 (0.86 – 0.97) | 40.8 | ± 0.90 x̄ |
| VLF (ms^2^) | 955.3 (636.7, 1794.3) | 984.8 (553.3, 1929.6) | 0.66 | 0.85 (0.70 – 0.93) | 48.5 | ± 1.01 x̄ |
| ULF (ms^2^) | 5196.8 (1938.6, 8051.1) | 3919.9 (2396.9, 6681.3) | 0.70 | 0.45 (0.09 – 0.71) | 56.2 | ± 1.10 x̄ |
| TP (ms^2^) | 7111.9 (4074.7, 12339.9) | 5505.9 (3610.7, 8998.5) | 0.73 | 0.66 (0.39 – 0.83) | 41.6 | ± 0.90 x̄ |
| 24-hr |  |  |  |  |  |  |
| SDNN (ms) | 98.2 (81.4, 119.3) | 108.0 (80.1, 125.5) | 0.75 | 0.69 (0.42 – 0.84) | 17.3 | ± 0.44 x̄ |
| RMSSD (ms) | 17.5 (12.1, 27.1) | 18.3 (11.3, 28.9) | 0.70 | 0.91 (0.81 – 0.96) | 15.7 | ± 0.40 x̄ |
| HF (ms^2^) | 210.9 (114.0, 417.6) | 243.4 (146.7, 438.6) | 0.31 | 0.89 (0.77 – 0.95) | 45.3 | ± 0.92 x̄ |
| LF (ms^2^) | 308.6 (202.8, 798.4) | 315.4 (237.0, 592.9) | 0.30 | 0.95 (0.90 – 0.98) | 26.3 | ± 0.60 x̄ |
| VLF (ms^2^) | 1141.2 (666.3, 1865.9) | 1194.9 (664.3, 1676.1) | 0.59 | 0.93 (0.85 – 0.97) | 21.1 | ± 0.53 x̄ |
| ULF (ms^2^) | 8226.7 (5480.3, 14421.5) | 10046.0 (5164.5, 14170.9) | 0.88 | 0.58 (0.26 – 0.79) | 46.3 | ± 0.98 x̄ |
| TP (ms^2^) | 9602.6 (6821.3, 16513.8) | 11745.3 (6745.2, 17532.1) | 0.97 | 0.69 (0.42 – 0.84) | 38.2 | ± 0.86 x̄ |

SDNN standard deviation of all normal-to-normal R-R intervals; RMSSD root mean square of successive differences between normal heartbeats; HF high frequency; LF low frequency; VLF very low frequency; ULF ultra-low frequency; TP total power; SD standard deviation; CI confidence interval; ICC intraclass correlation coefficient; CV coefficient of variation; LOA limits of agreement; x̄ data mean

**Supplementary table 3** Test-retest reliability of HRV for each duration (Tetraplegia, n = 11)

|  | Day 1,  median (p25, p75) | Day 2,  median (p25, p75) | p-value | ICC (95%CI) | CV | 95% LoA |
| --- | --- | --- | --- | --- | --- | --- |
| 5-min |  |  |  |  |  |  |
| SDNN (ms) | 50.7 (28.8, 72.0) | 33.9 (25.3, 55.3) | 0.41 | 0.22 (-0.34 - 0.70) | 62.2 | ± 1.12 x̄ |
| RMSSD (ms) | 18.3 (10.4, 33.7) | 16.9 (11.5, 23.3) | 0.37 | 0.43 (-0.14 - 0.80) | 57.1 | ± 1.08 x̄ |
| HF (ms^2^) | 180.0 (51.6, 395.6) | 90.9 (39.3, 171.4) | 0.52 | 0.34 (-0.28 - 0.77) | 163.0 | ± 1.70 x̄ |
| LF (ms^2^) | 439.9 (119.4, 861.9) | 190.3 (124.7, 809.3) | 0.83 | 0.17 (-0.49 - 0.68) | 112.6 | ± 1.55 x̄ |
| TP (ms^2^) | 619.9 (142.8, 1562.1) | 272.1 (149.4, 980.7) | 0.76 | 0.24 (-0.40 - 0.72) | 129.2 | ± 1.62 x̄ |
| 10-min |  |  |  |  |  |  |
| SDNN (ms) | 56.6 (41.2, 72.2) | 42.9 (22.5, 51.9) | 0.17 | 0.28 (-0.24 - 0.72) | 51.6 | ± 0.89 x̄ |
| RMSSD (ms) | 16.3 (9.8, 36.0) | 17.7 (11.8, 23.1) | 0.83 | 0.60 (0.05 - 0.87) | 43.8 | ± 0.93 x̄ |
| HF (ms^2^) | 143.7 (35.2, 334.8) | 114.6 (42.2, 453.2) | 0.70 | 0.50 (-0.15 - 0.84) | 111.2 | ± 1.56 x̄ |
| LF (ms^2^) | 372.5 (83.9, 704.4) | 189.8 (89.6, 772.6) | 0.64 | 0.41 (-0.28 - 0.80) | 81.5 | ± 1.34 x̄ |
| VLF (ms^2^) | 2097.3 (936.0, 3630.7) | 1274.4 (430.6, 1937.0) | 0.12 | 0.02 (-0.50 - 0.58) | 154.4 | ± 1.57 x̄ |
| TP (ms^2^) | 2601.0 (1664.5, 4852.1) | 1654.4 (496.1, 2903.5) | 0.24 | 0.06 (-0.49 - 0.60) | 136.5 | ± 1.52 x̄ |
| 1-hr |  |  |  |  |  |  |
| SDNN (ms) | 64.5 (57.2, 92.4) | 50.9 (42.1, 67.4) | 0.17 | 0.60 (0.09 – 0.87) | 27.3 | ± 0.60 x̄ |
| RMSSD (ms) | 19.1 (13.4, 37.3) | 18.9 (14.4, 25.1) | 0.32 | 0.71 (0.20 – 0.91) | 32.4 | ± 0.77 x̄ |
| HF (ms^2^) | 127.4 (86.1, 297.4) | 224.0 (114.9, 496.5) | 0.08 | 0.53 (-0.03 – 0.85) | 98.8 | ± 1.47 x̄ |
| LF (ms^2^) | 257.1 (73.8, 611.4) | 251.5 (174.5, 739.5) | 0.15 | 0.73 (0.28 – 0.92) | 69.9 | ± 1.24 x̄ |
| VLF (ms^2^) | 1058.1 (612.4, 2597.8) | 791.5 (516.1, 1992.1) | 1.00 | 0.70 (0.19 – 0.91) | 44.7 | ± 0.96 x̄ |
| ULF (ms^2^) | 2671.4 (1687.9, 5454.9) | 1356.1 (871.6, 1755.3) | 0.007 | 0.52 (-0.07 – 0.85) | 100.9 | ± 1.33 x̄ |
| TP (ms^2^) | 4565.9 (3290.9, 9698.7) | 2683.0 (1717.7, 5082.2) | 0.21 | 0.68 (0.20 – 0.90) | 63.0 | ± 1.11 x̄ |
| 3-hr |  |  |  |  |  |  |
| SDNN (ms) | 73.4 (58.8, 112.4) | 74.4 (52.4, 98.5) | 0.37 | 0.65 (0.16 - 0.89) | 24.1 | ± 0.54 x̄ |
| RMSSD (ms) | 22.7 (14.7, 37.8) | 23.3 (15.3, 27.1) | 0.17 | 0.46 (-0.19 – 0.82) | 40.0 | ± 0.90 x̄ |
| HF (ms^2^) | 213.5 (100.2, 379.4) | 324.6 (89.4, 710.1) | 0.12 | 0.20 (-0.48 – 0.71) | 137.1 | ± 1.68 x̄ |
| LF (ms^2^) | 327.7 (135.9, 842.6) | 403.1 (129.3, 1247.8) | 0.17 | 0.37 (-0.30 – 0.78) | 99.6 | ± 1.51 x̄ |
| VLF (ms^2^) | 1135.9 (616.5, 2900.7) | 787.3 (459.7, 2750.4) | 0.70 | 0.66 (0.12 – 0.90) | 52.0 | ± 1.06 x̄ |
| ULF (ms^2^) | 3305.9 (2492.1, 9506.2) | 2332.4 (1778.0, 6075.1) | 0.17 | 0.53 (-0.01 – 0.84) | 89.9 | ± 1.25 x̄ |
| TP (ms^2^) | 5764.6 (3534.9, 12840.1) | 5955.0 (2870.3, 9925.8) | 0.52 | 0.54 (-0.001 – 0.85) | 55.9 | ± 1.05 x̄ |
| 6-hr |  |  |  |  |  |  |
| SDNN (ms) | 74.1 (62.1, 111.4) | 79.8 (55.2, 110.9) | 0.41 | 0.79 (0.40 – 0.94) | 16.0 | ± 0.41 x̄ |
| RMSSD (ms) | 22.3 (13.3, 35.9) | 24.2 (13.2, 29.2) | 1.00 | 0.83 (0.51 – 0.95) | 17.3 | ± 0.45 x̄ |
| HF (ms^2^) | 182.1 (131.5, 442.3) | 303.2 (163.3, 550.8) | 0.21 | 0.57 (-0.05 – 0.86) | 63.3 | ± 1.16 x̄ |
| LF (ms^2^) | 277.5 (130.2, 792.9) | 247.8 (162.5, 875.5) | 0.52 | 0.64 (0.09 – 0.89) | 50.5 | ± 1.04 x̄ |
| VLF (ms^2^) | 1311.7 (498.9, 2272.0) | 726.1 (417.3, 2495.1) | 0.37 | 0.86 (0.55 – 0.96) | 24.9 | ± 0.60 x̄ |
| ULF (ms^2^) | 3494.1 (2945.3, 11411.0) | 4855.0 (2243.0, 8172.9) | 0.41 | 0.69 (0.20 – 0.90) | 53.4 | ± 1.08 x̄ |
| TP (ms^2^) | 5869.3 (3893.1, 12915.1) | 6787.7 (3006.1, 13386.0) | 0.46 | 0.69 (0.19 – 0.91) | 36.3 | ± 0.82 x̄ |
| 24-hr |  |  |  |  |  |  |
| SDNN (ms) | 97.6 (73.6, 130.3) | 109.4 (70.0, 126.4) | 0.52 | 0.83 (0.50 – 0.95) | 13.3 | ± 0.35 x̄ |
| RMSSD (ms) | 31.2 (15.6, 35.3) | 30.0 (15.6, 34.0) | 0.41 | 0.91 (0.72 – 0.98) | 11.6 | ± 0.31 x̄ |
| HF (ms^2^) | 403.4 (156.1, 757.2) | 439.8 (171.5, 522.5) | 0.76 | 0.51 (-0.13 – 0.84) | 43.7 | ± 0.96 x̄ |
| LF (ms^2^) | 321.0 (211.2, 714.9) | 335.9 (165.9, 974.1) | 0.76 | 0.66 (0.11 – 0.90) | 44.2 | ± 0.97 x̄ |
| VLF (ms^2^) | 1298.6 (599.3, 2910.8) | 1197.6 (491.2, 2148.8) | 0.15 | 0.87 (0.60 – 0.96) | 22.3 | ± 0.49 x̄ |
| ULF (ms^2^) | 6317.5 (4841.8, 13848.1) | 10046.0 (4479.2, 13693.9) | 0.70 | 0.71 (0.24 – 0.91) | 39.5 | ± 0.88 x̄ |
| TP (ms^2^) | 10598.9 (6199.0, 17380.4) | 11745.3 (5092.0, 16734.7) | 0.70 | 0.74 (0.30 – 0.92) | 29.7 | ± 0.72 x̄ |

SDNN standard deviation of all normal-to-normal R-R intervals; RMSSD root mean square of successive differences between normal heartbeats; HF high frequency; LF low frequency; VLF very low frequency; ULF ultra-low frequency; TP total power; SD standard deviation; CI confidence interval; ICC intraclass correlation coefficient; CV coefficient of variation; LOA limits of agreement; x̄ data mean

**Supplementary table 4** Test-retest reliability of HRV for each duration (Paraplegia, n = 34)

|  | Day 1,  median (p25, p75) | Day 2,  median (p25, p75) | p-value | ICC (95%CI) | CV | 95% LoA |
| --- | --- | --- | --- | --- | --- | --- |
| 5-min |  |  |  |  |  |  |
| SDNN (ms) | 44.5 (29.8, 53.0) | 41.6 (30.1, 61.0) | 0.26 | 0.38 (0.05 - 0.64) | 44.0 | ± 0.94 x̄ |
| RMSSD (ms) | 15.2 (10.3, 28.3) | 18.0 (8.7, 26.5) | 0.95 | 0.84 (0.71 - 0.92) | 38.9 | ± 0.86 x̄ |
| HF (ms^2^) | 109.6 (52.9, 399.7) | 144.5 (45.1, 441.9) | 0.91 | 0.67 (0.44 - 0.82) | 132.8 | ± 1.66 x̄ |
| LF (ms^2^) | 508.0 (206.5, 773.7) | 324.7 (147.5, 780.6) | 0.45 | 0.45 (0.14 - 0.68) | 102.0 | ± 1.51 x̄ |
| TP (ms^2^) | 672.8 (236.2, 1105.9) | 487.9 (214.7, 1142.8) | 0.88 | 0.58 (0.31 - 0.77) | 90.8 | ± 1.44 x̄ |
| 10-min |  |  |  |  |  |  |
| SDNN (ms) | 50.5 (32.1, 68.1) | 46.1 (33.9, 68.7) | 0.96 | 0.48 (0.17 - 0.70) | 48.5 | ± 1.01 x̄ |
| RMSSD (ms) | 16.4 (9.6, 27.9) | 19.6 (8.9, 25.3) | 0.61 | 0.82 (0.66 - 0.90) | 39.5 | ± 0.87 x̄ |
| HF (ms^2^) | 143.0 (56.8, 419.1) | 133.2 (70.4, 364.4) | 0.77 | 0.72 (0.50 - 0.85) | 138.7 | ± 1.67 x̄ |
| LF (ms^2^) | 477.9 (208.1, 663.4) | 298.7 (178.4, 603.6) | 0.022 | 0.60 (0.33 - 0.78) | 96.8 | ± 1.46 x̄ |
| VLF (ms^2^) | 1526.4 (733.0, 3269.5) | 1351.5 (618.4, 3223.2) | 0.87 | 0.22 (-0.13 - 0.52) | 140.7 | ± 1.69 x̄ |
| TP (ms^2^) | 2252.6 (1072.7, 4690.4) | 1857.7 (944.2, 4642.5) | 0.84 | 0.27 (-0.07 - 0.56) | 121.9 | ± 1.62 x̄ |
| 1-hr |  |  |  |  |  |  |
| SDNN (ms) | 61.5 (47.9, 89.0) | 66.4 (45.3, 92.1) | 0.20 | 0.43 (0.11– 0.67) | 44.2 | ± 0.76 x̄ |
| RMSSD (ms) | 16.9 (10.2, 29.3) | 17.3 (8.4, 29.0) | 0.48 | 0.76 (0.57 – 0.87) | 51.9 | ± 0.93 x̄ |
| HF (ms^2^) | 165.4 60.2, 384.1) | 163.3 (71.0, 316.3) | 0.81 | 0.66 (0.42 – 0.81) | 114.1 | ± 1.50 x̄ |
| LF (ms^2^) | 326.9 (143.4, 768.7) | 302.1 (207.4, 622.2) | 0.66 | 0.77 (0.58 – 0.88) | 101.4 | ± 1.23 x̄ |
| VLF (ms^2^) | 1152.0 (920.0, 1887.1) | 1238.5 (791.5, 3316.2) | 0.23 | 0.63 (0.38 – 0.79) | 83.7 | ± 1.15 x̄ |
| ULF (ms^2^) | 2182.5 (1001.3, 4511.7) | 2755.7 (1454.6, 5057.5) | 0.25 | 0.02 (-0.33 – 0.36) | 177.0 | ± 1.64 x̄ |
| TP (ms^2^) | 4039.3 (2471.4, 7979.6) | 5263.8 (2285.7, 9340.5) | 0.11 | 0.25 (-0.11 – 0.54) | 108.2 | ± 1.34 x̄ |
| 3-hr |  |  |  |  |  |  |
| SDNN (ms) | 81.2 (63.8, 109.7) | 76.7 (63.5, 99.0) | 0.81 | 0.74 (0.53 - 0.86) | 22.7 | ± 0.56 x̄ |
| RMSSD (ms) | 17.3 (11.1, 28.2) | 15.2 (9.0, 25.2) | 0.31 | 0.89 (0.79 – 0.94) | 34.3 | ± 0.78 x̄ |
| HF (ms^2^) | 221.6 (105.9, 372.0) | 123.7 (70.4, 402.5) | 0.06 | 0.81 (0.66 – 0.90) | 80.3 | ± 1.34 x̄ |
| LF (ms^2^) | 297.2 (209.6, 628.5) | 264.5 (169.6, 562.9) | 0.16 | 0.92 (0.85 – 0.96) | 51.4 | ± 1.04 x̄ |
| VLF (ms^2^) | 1121.4 (681.7, 1794.3) | 992.5 (728.1, 1929.6) | 0.73 | 0.81 (0.66 – 0.90) | 52.9 | ± 1.06 x̄ |
| ULF (ms^2^) | 5271.2 (2448.5, 9005.4) | 4468.1 (2598.6, 8392.6) | 0.71 | 0.71 (0.49 – 0.84) | 73.9 | ± 1.30 x̄ |
| TP (ms^2^) | 7290.1 (4074.7, 12474.0) | 6124.1 (4055.4, 10284.9) | 0.69 | 0.75 (0.56 – 0.87) | 52.4 | ± 1.06 x̄ |
| 6-hr |  |  |  |  |  |  |
| SDNN (ms) | 85.0 (68.2, 103.3) | 78.3 (64.1, 106.8) | 0.45 | 0.84 (0.70 – 0.92) | 17.3 | ± 0.44 x̄ |
| RMSSD (ms) | 16.0 (10.1, 29.5) | 15.3 (9.7, 26.8) | 0.44 | 0.91 (0.82 – 0.95) | 28.1 | ± 0.69 x̄ |
| HF (ms^2^) | 162.0 (89.7, 331.9) | 140.4 (81.1, 327.3) | 0.13 | 0.86 (0.73 – 0.93) | 49.4 | ± 1.02 x̄ |
| LF (ms^2^) | 256.9 (184.0, 652.6) | 277.1 (159.6, 625.6) | 0.64 | 0.93 (0.87 – 0.97) | 37.1 | ± 0.83 x̄ |
| VLF (ms^2^) | 1118.6 (568.9, 1821.0) | 940.3 (641.9, 1557.6) | 0.79 | 0.86 (0.74 – 0.93) | 43.2 | ± 0.93 x̄ |
| ULF (ms^2^) | 6079.8 (3656.4, 8516.8) | 4745.3 (2901.3, 8651.3) | 0.50 | 0.82 (0.68 – 0.91) | 53.1 | ± 1.06 x̄ |
| TP (ms^2^) | 7857.2 (4481.9, 11874.8) | 6276.9 (4102.5, 11374.6) | 0.45 | 0.87 (0.75 – 0.93) | 39.8 | ± 0.87 x̄ |
| 24-hr |  |  |  |  |  |  |
| SDNN (ms) | 102.9 (81.5, 132.5) | 108.5 (82.2, 128.0) | 0.99 | 0.82 (0.67 – 0.91) | 16.4 | ± 0.42 x̄ |
| RMSSD (ms) | 18.9 (12.2, 34.8) | 21.5 (11.3, 32.6) | 0.67 | 0.92 (0.84 – 0.96) | 15.9 | ± 0.41 x̄ |
| HF (ms^2^) | 249.6 (118.9, 563.1) | 277.0 (146.7, 474.3) | 0.58 | 0.89 (0.80 – 0.95) | 41.9 | ± 0.89 x̄ |
| LF (ms^2^) | 339.8 (211.2, 933.0) | 396.5 (250.0, 839.4) | 0.75 | 0.93 (0.87 – 0.97) | 25.1 | ± 0.60 x̄ |
| VLF (ms^2^) | 1271.5 (729.4, 1865.9) | 1251.3 (799.5, 1765.3) | 0.85 | 0.94 (0.93 – 0.97) | 20.6 | ± 0.51 x̄ |
| ULF (ms^2^) | 8772.8 (5522.5, 14542.9) | 9731.9 (5235.0, 14909.1) | 0.59 | 0.83 (0.69 – 0.91) | 43.4 | ± 0.94 x̄ |
| TP (ms^2^) | 11014.7 (7052.4, 18311.4) | 12078.2 (6949.5, 18282.0) | 0.71 | 0.86 (0.74 – 0.93) | 36.3 | ± 0.82 x̄ |

SDNN standard deviation of all normal-to-normal R-R intervals; RMSSD root mean square of successive differences between normal heartbeats; HF high frequency; LF low frequency; VLF very low frequency; ULF ultra-low frequency; TP total power; SD standard deviation; CI confidence interval; ICC intraclass correlation coefficient; CV coefficient of variation; LOA limits of agreement; x̄ data mean
